# Supplementary material for: Carbon-Ion Radiotherapy for Prostate Cancer in Patients with a History of Surgery for Benign Prostatic Hyperplasia
Source: Cancers (Basel). 2025 Sep 17;17(18):3039. doi: 10.3390/cancers17183039 (PMC12469118; doi:10.3390/cancers17183039)
Supplement: Supplementary file 1 [file cancers-17-03039-s001.zip › cancers-3828742-supplementary.pdf]

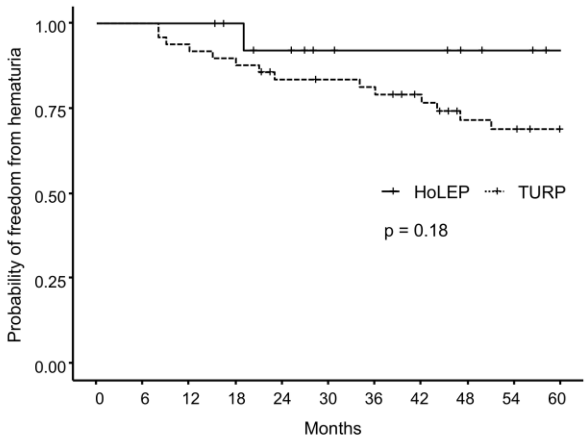

Figure S1. Kaplan–Meier curves of freedom from hematuria stratified by surgical modality (TURP vs. HoLEP). No significant difference was observed (log-rank $p = 0.18$).

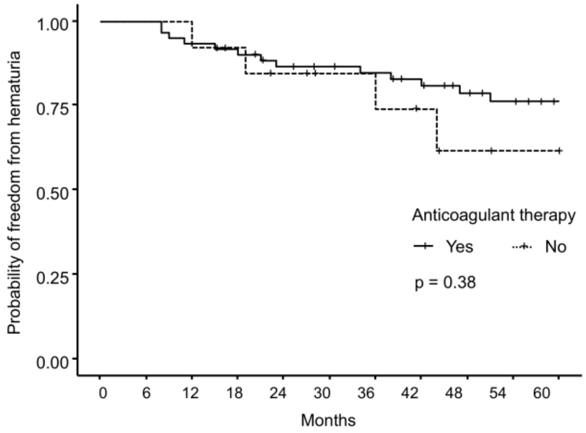

Figure S2. Kaplan–Meier curves of freedom from hematuria stratified by anticoagulant therapy (use vs. non-use). No significant difference was observed (log-rank $p = 0.38$).
